# Preventive Hyperbaric Oxygen Therapy for Asymptomatic Left Ventricular Air During CT-Guided Lung Needle Biopsy

**DOI:** 10.7759/cureus.55665

**Published:** 2024-03-06

**Authors:** Natsuki Kondo, Shinya Suzuki

**Affiliations:** 1 Emergency Medicine, Koga Community Hospital, Yaizu, JPN; 2 Emergency and Trauma Care, Kameda Medical Center, Kamogawa, JPN

**Keywords:** hyperbaric oxygen therapy (hbot), lung biopsy, collaboration with other medical departments, rare complications, arterial gas embolism, u.s. navy table 5

## Abstract

Lung needle biopsy can cause air to enter the vessels due to the traffic between the vessels and the trachea. Hyperbaric oxygen therapy (HBOT) according to the U.S. Navy Treatment Table (USNTT) 6 or 6A protocol is used for arterial gas embolism (AGE). However, no treatment or HBOT protocol for asymptomatic intra-arterial air has been established. Here we report two cases of asymptomatic intra-arterial air during lung needle biopsy that were preventively treated with HBOT according to the USNTT 5 protocol. In case 1, a 72-year-old man with malignant lymphoma in remission underwent computed tomography (CT)-guided lung needle biopsy of a nodule in his right lung. During the biopsy, the patient developed a cough, followed by chest pain and dyspnea. Chest CT revealed a right pneumothorax and air in the left ventricle and aorta. The patient did not present with symptoms suggestive of AGE. After thoracic drainage, 4.5 hours after onset, the patient underwent HBOT according to the USNTT 5 protocol. After one session in the hyperbaric chamber, follow-up whole-body CT showed disappearance of intravascular air. In case 2, a 69-year-old man with chronic obstructive pulmonary disease underwent CT-guided lung needle biopsy of a nodule in his right lung. Post-examination CT showed intravascular air in the aorta, pulmonary artery and vein, and left ventricle. However, the patient had no symptoms. One hour after onset, the patient underwent HBOT according to the USNTT 5 protocol. A whole-body CT the next day confirmed the disappearance of intravascular air. Both patients were discharged without sequelae. HBOT is an effective treatment to flush out intra-arterial air and inhibit the expression of adhesion molecules. Asymptomatic intra-arterial air may be adequately treated with HBOT according to a short protocol such as USNTT 5.

## Introduction

Arterial air embolism (AGE) is a rare but serious complication of lung needle biopsy. Most lung needle biopsies are performed under computed tomography (CT) guidance; however, AGE can also occur in CT-guided lung needle biopsies [1,2]. Hyperbaric oxygen therapy (HBOT) according to the U.S. Navy Treatment Table (USNTT) 6 or 6A protocol is recommended for the treatment of AGE [3].

Some cases have reported HBOT performed for AGE occurring during lung needle biopsy with good outcomes [1,2]. However, the efficacy of HBOT in preventing the development of asymptomatic intra-arterial air is not yet known, and the optimal treatment protocol has not been established. This report describes two successful cases of asymptomatic intra-arterial air that developed during CT-guided lung needle biopsy which was treated with preventive HBOT according to the USNTT 5 protocol.

## Case presentation

Case 1

**Figure 1:**
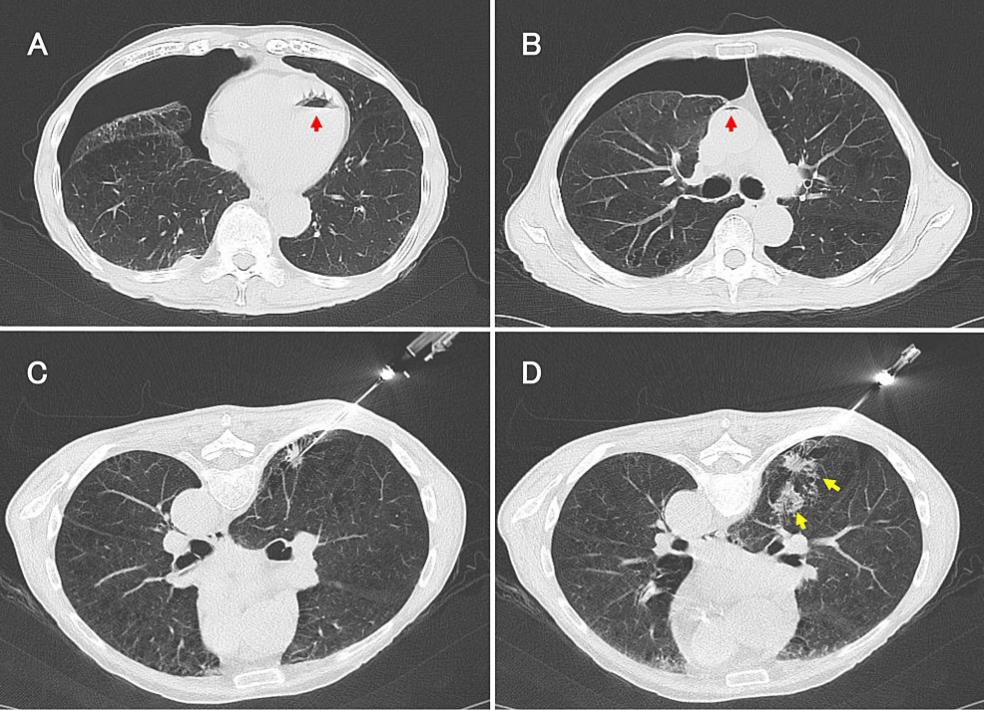
Chest CT images during CT-guided lung needle biopsy in case 1. Axial chest computed tomography images post lung needle biopsy, showing air within the left ventricle (A) and aorta (B). At the beginning of the examination, there was no bleeding around the nodule (C); however, bleeding occurred during the examination (D). Red arrow: intravascular air. Yellow arrow: bleeding.

A 72-year-old man with malignant lymphoma in remission was admitted for evaluation of right-sided back pain. Chest CT detected a small nodule in his right lung, and a lung biopsy was assembled. During the biopsy, the patient coughed and presented with chest pain and dyspnea. Subsequent whole-body CT showed a right pneumothorax and air in the left ventricle (Figure 1A) and aorta (Figure 1B). While no bleeding was initially observed (Figure 1C), bleeding around the puncture site occurred during the biopsy (Figure 1D).

**Figure 2:**
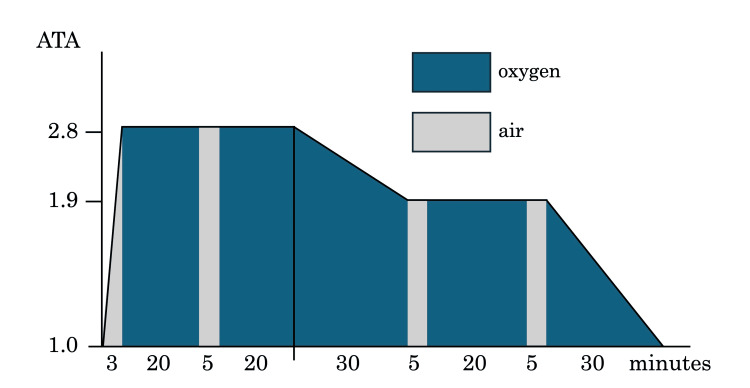
The U.S. Navy Treatment Table 5 protocol of hyperbaric oxygen therapy. Air was pressurized to 2.8 ATA over 3 minutes and pure oxygen was intermittently administered at 20-minute intervals. The pressure was then reduced to 1.9 ATA over 30 minutes, and pure oxygen was administered for 20 minutes. The pressure was then depressurized to 1 ATA over 30 minutes. The total duration of hyperbaric oxygen therapy was 2 hours 18 minutes. ATA: Atmosphere Absolute

Considering the risk of intra-arterial air embolism with movement, the patient was placed in the right lateral recumbent position and received 100% high-flow oxygen and thoracic drainage. Ninety minutes of bed rest did not produce any embolic symptoms; however, re-scanned CT showed residual air in the left ventricle. As it was asymptomatic intra-arterial air, the patient received hyperbaric oxygen therapy according to the USNTT 5 protocol 4.5 hours after onset (Figure 2). After one session in the hyperbaric chamber, a follow-up whole-body CT revealed the disappearance of intravascular air. The patient was discharged without complications, and head magnetic resonance imaging showed no findings of infarction.

Case 2

**Figure 3:**
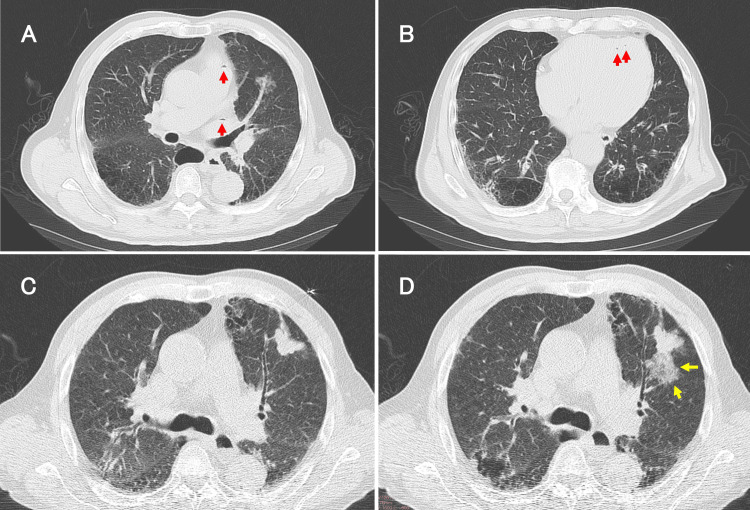
Chest CT images during CT-guided lung needle biopsy in case 2. Axial chest computed tomography images post lung needle biopsy, showing air within the pulmonary artery and vein (A) and left ventricle (B). At the beginning of the examination, there was no bleeding around the nodule (C); however, bleeding occurred during the examination (D). Red arrow: intravascular air. Yellow arrow: bleeding.

A 69-year-old man with chronic obstructive pulmonary disease (COPD) was found to have an abnormal chest radiograph during a medical checkup. A chest CT revealed a small nodule in the left lung, and the patient was admitted for a CT-guided lung needle biopsy. During the biopsy, the patient was asymptomatic. After the examination, chest CT revealed intravascular air in the pulmonary artery and vein (Figure 3A), and left ventricle (Figure 3B). While no bleeding was initially observed (Figure 3C), bleeding around the puncture site occurred during the biopsy (Figure 3D). Head CT showed no obvious air. The patient received oxygen and, one hour after onset, underwent HBOT according to the USNTT 5 protocol. The patient was admitted to the intensive care unit after one session. Whole-body CT the next day revealed disappearance of intravascular air. The patient was discharged without sequelae.

## Discussion

The incidence of systemic AGE from lung needle biopsy has been reported to be 0.02-0.07% [4,5] and has traditionally been considered a rare complication. Recently, however, a rate of about 0.21% [6] has been reported, suggesting that it may occur more frequently. Two mechanisms have been postulated to cause intra-arterial air in lung needle biopsies: the first is the influx of air from outside the thoracic cavity into the pulmonary veins [7]. Risk factors include the number of punctures and increased intrathoracic pressure such as coughing or breath holding. The second is direct traffic between the airway system and the pulmonary venous system [8]. Bleeding on puncture and biopsy of cystic or cavitary lesions are also risk factors [9]. Certain diseases, such as coagulation abnormalities, inflammation, vasculitis, and fragile lung tissue, can also cause prolonged exposure of the airway to vascular air, where the normal hemostatic mechanisms do not work well [10]. In case 1, the increased intrathoracic pressure caused by coughing was responsible for the influx of air from outside the thoracic cavity into the pulmonary veins and the direct traffic between the airway system and the pulmonary veins, while in case 2, the medical history of COPD and direct traffic between the airway system and pulmonary veins were involved.

Intra-arterial air can cause embolism as well as ischemia-reperfusion injury. Intra-arterial air induces activation of vascular endothelial cells and expression of adhesion molecules [3]. It leads to platelet aggregation around air bubbles with activation of neutrophils and inflammatory changes, causing capillary stenosis and vascular endothelial damage. This may result in increased vascular permeability, acute respiratory distress syndrome, impaired consciousness, and seizures [3]. Although all of the present cases were asymptomatic intra-arterial air, residual air could cause progressive or delayed ischemia-reperfusion injury. HBOT was performed to flush out intraventricular and intra-arterial air and to inhibit the expression of adhesion molecules.

HBOT is the only effective treatment for AGE. Only a limited number of centers are able to perform HBOT, even though lung biopsy is available. Cases of patients with intra-arterial air have been reported without HBOT [11]. However, the safe threshold for intra-arterial air remains uncertain. It is also difficult to accurately estimate the volume of intravascular air on CT. Even in hospitals where HBOT can be performed, it is advisable to administer 100% high-flow oxygen and to position the patient in the right lateral decubitus position as soon as intra-arterial air is detected. The administration of high-flow oxygen flushes out inactive gases and improves tissue hypoxia [12]. In the right-sided supine position, the left ventricular outflow tract is inferior, allowing air in the left ventricle to remain upward in a nondependent manner, away from the aorta, thus preventing air from entering the cerebral and systemic circulation. The Trendelenburg position is currently not recommended due to its poor effectiveness and potential to exacerbate cerebral edema [13].

When intra-arterial air migrates into the cerebral vasculature and causes embolism, a link between time to initiation of HBO and neurologic prognosis has been noted [14]. The time allowed from the onset of arterial air embolism to initiation of HBOT is unknown. Prompt HBOT is advisable when intra-arterial air is recognized. HBOT with the USNTT 6 or 6A protocol is recommended for arterial air embolism [3]. However, both protocols are long table HBOT with treatment times of approximately 5-6 hours, and prolonged treatment or hyperbaric treatment may result in oxygen toxicity or barotrauma [3]. Our choice of HBOT protocols is based on the fact that the HBOT protocol is not a standardized one. The USNTT 5 protocol we selected is a short table HBOT, indicated for mild decompression sickness, with a treatment time of approximately 2.5 hours and has a lower risk or threshold for treatment. No arterial embolic symptoms or ischemia-reperfusion injury occurred in both cases. In asymptomatic intra-arterial air, short table HBOT may be sufficient.

## Conclusions

The standard protocol for AGE is long table HBOT such as USNTT 6 or 6A. In asymptomatic patients, however, short table HBOT such as USNTT 5 may be sufficient to prevent the development of AGE and ischemia-reperfusion injury. If intra-arterial air occurs during lung needle biopsy, early initiation of HBOT may be recommended to prevent the onset of AGE. Hospitals lacking HBOT facilities should consider establishing collaborative networks with institutions equipped to provide HBOT, particularly during lung needle biopsy procedures.

## Figures and Tables

**Figure 4:**
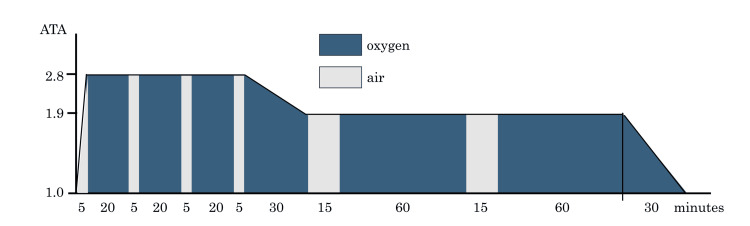
The U.S. Navy Treatment Table 6 protocol of hyperbaric oxygen therapy. Air was pressurized to 2.8 ATA over 5 minutes while pure oxygen was administered intermittently at 20-minute intervals. It was then depressurized to 1.9 ATA over 30 minutes, and pure oxygen was again administered intermittently at 60-minute intervals. The pressure was then reduced to 1 ATA over 30 minutes. The total duration of hyperbaric oxygen therapy was 4 hours 50 minutes. ATA: Atmosphere Absolute

**Figure 5:**
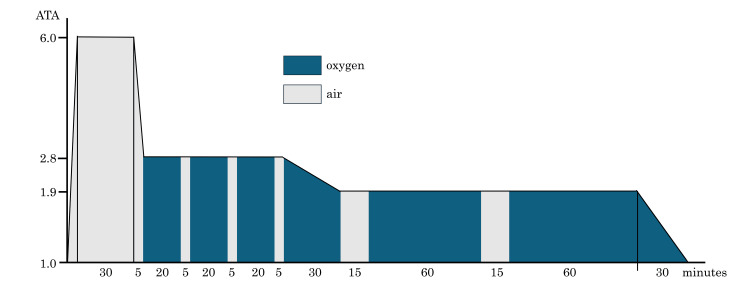
The U.S. Navy Treatment Table 6A protocol of hyperbaric oxygen therapy. Air was pressurized to 6 ATA and this pressure was maintained for 30 minutes, then reduced to 2.8 ATA, and maintained for 5 minutes, and pure oxygen was administered intermittently at 20-minute intervals. The pressure was then reduced to 1.9 ATA over 30 minutes. This pressure was maintained for 150 minutes while pure oxygen was administered intermittently at 60-minute intervals. The pressure was then reduced to 1 ATA over 30 minutes. The total duration of standard hyperbaric oxygen therapy was 5 hours and 30 minutes. ATA: Atmosphere Absolute
